# The Pleiotropic Effects of Fumarate: From Mitochondrial Respiration to Epigenetic Rewiring and DNA Repair Mechanisms

**DOI:** 10.3390/metabo13070880

**Published:** 2023-07-24

**Authors:** Sebastiano Giallongo, Francesco Costa, Lucia Longhitano, Cesarina Giallongo, Jessica Ferrigno, Emanuela Tropea, Nunzio Vicario, Giovanni Li Volti, Rosalba Parenti, Ignazio Barbagallo, Vincenzo Bramanti, Daniele Tibullo

**Affiliations:** 1 Department of Biomedical and Biotechnological Sciences, University of Catania, 95123 Catania, Italy; sebastiano.giall@gmail.com (S.G.); frank92costa@gmail.com (F.C.); lucia.longhitano@unict.it (L.L.); jessica.ferrigno.1996@gmail.com (J.F.); tropeaemanuela3@gmail.com (E.T.); nunziovicario@unict.it (N.V.); livolti@unict.it (G.L.V.); parenti@unict.it (R.P.); ignazio.barbagallo@unict.it (I.B.); 2 Department of Medical-Surgical Science and Advanced Technologies “Ingrassia”, University of Catania, 95123 Catania, Italy; cesarina.giallongo@unict.it; 3 U.O.C. Laboratory Analysis, ASP Ragusa, 97100 Ragusa, Italy; vincenzo.bramanti@asp.rg.it

**Keywords:** metabolism, fumarate, epigenetics, methylation, DNA repair

## Abstract

Tumor onset and its progression are strictly linked to its metabolic rewiring on the basis of the Warburg effect. In this context, fumarate emerged as a putative oncometabolite mediating cancer progression. Fumarate accumulation is usually driven by fumarate hydratase (FH) loss of function, the enzyme responsible for the reversible conversion of fumarate into malate. Fumarate accumulation acts as a double edge sword: on one hand it takes part in the metabolic rewiring of cancer cells, while on the other it also plays a crucial role in chromatin architecture reorganization. The latter is achieved by competing with a-ketoglutarate-dependent enzymes, eventually altering the cellular methylome profile, which in turn leads to its transcriptome modeling. Furthermore, in recent years, it has emerged that FH has an ability to recruit DNA double strand breaks. The accumulation of fumarate into damaged sites might also determine the DNA repair pathway in charge for the seizure of the lesion, eventually affecting the mutational state of the cells. In this work, we aimed to review the current knowledge on the role of fumarate as an oncometabolite orchestrating the cellular epigenetic landscape and DNA repair machinery.

## 1. Fumarate Accumulation Drives Tumorigenesis

### 1.1. Fumarate Metabolism and Accumulation

**Figure 1:**
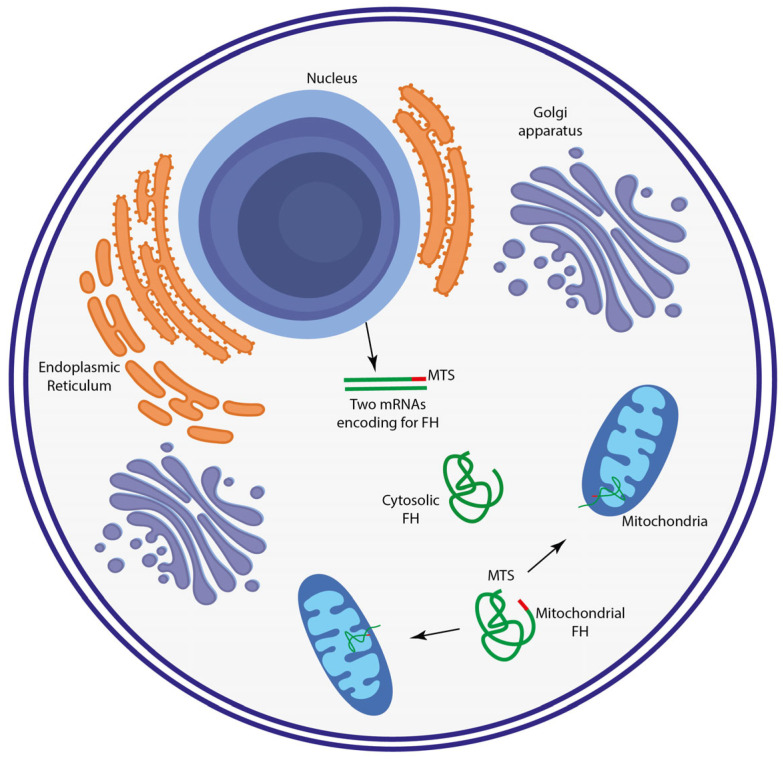
Representative image showing the shuttle of FH transcripts within different cellular compartments. FH is transcribed by two different genes encoding for the cytosolic and the mitochondrial isoform. The latter harbors an MTS, determining its mitochondrial translocation.

The typical metabolic rewiring of cancer cells has been reported as an emerging hallmark of cancers, potentially representing a valid target to be exploited for the development of novel therapies [1,2]. To date, it is widely accepted that metabolic reprograming is crucial in sustaining the biosynthetic needs underneath tumor progression. Within this context, several mutations have been identified, eventually leading to the accumulation of different metabolites which have been investigated as potential factors regulating tumor progression. Among them, fumarate, an intermediate of Tricarboxylic Acid Cycle (TCA), has been reported as a critical metabolite within the cancerous context [3]. Alongside TCA, fumarate is converted to malate by the reversible hydration, catalyzed by fumarate hydratase (FH) [3]. This enzyme has been reported to harbor two isoforms: the first one located in the mitochondria serving for TCA, while the second one is in the cytosol where it takes part in purine and urea cycles. Interestingly, the mechanism orchestrating FH localization within the cellular compartments is still a matter of debate. This hypothesis is based on the cleavage of the FH pro-peptide into two smaller peptides, one of which harbors the N-terminal Mitochondrial Targeting Sequence (MTS), and it is hence shuttled to this organelle, which is likely the most realistic option (Figure 1) [4].

FH activity is therefore strictly linked to fumarate levels, which have been reported to be increased in different pathological conditions. Interestingly, FH deficiency has been detected to account for neonatal and early infantile encephalopathy characterized by lethargy, seizure and hypotonia [5]. This condition, also named fumaric aciduria, leads to a wide spectrum of clinical manifestation, but to date it has been poorly characterized given the low incidence: around 40 cases have been reported in the literature in the last 30 years. From a biochemical point of view, these patients show a marked increase in fumarate, eventually leading to a survival rate of 2 years of life upon diagnosis [5].

Further evidence also linked FH deficiency to tumorigenesis. Within the tumoral context, several reports claimed indeed that FH might carry mutations triggering fumarate accumulation. This scenario is typical of Hereditary Leiomyomatosis and Renal Cell Carcinoma (HLRCC), where the FH gene is affected by mutations or chromosomal aberrations at the 1p43 locus [6]. FH mutations are not the only factor triggering fumarate accumulation, as it is also correlated with the loss of function of enzymes such as Isocitrate Dehydrogenase 1 and 2 (IDH 1/2) and Succinate Dehydrogenase (SDH) [7]. Further evidence has associated FH impairment with a metabolic shift from Oxidative Phosphorylation (OXPHOS) towards glycolysis by decreasing the levels of the master metabolic regulator AMP-mediated protein kinase (AMPK), as described by the Warburg Effect [8]. Following its accumulation, fumarate might hamper SDH activity. Since SDH is part of the Complex II of the respiratory chain, this triggers the drastic reduction of mitochondrial respiration, thus promoting the metabolic shift towards glycolysis [9]. This outcome is enforced by the inhibition of pyruvate dehydrogenase (PDH), in turn blocking pyruvate influx into mitochondria. The mechanism orchestrating PDH blockage is linked to fumarate accumulation, which acts as a competitive inhibitor of Prolyl Hydroxylases (PHDs), a 2OG-dependent oxygenase, leading to the stabilization of the Hypoxia-inducible Factor 1/2-alpha (HIF1-α/HIF2-α) and eventually triggering the expression of Pyruvate Dehydrogenase Kinase 1 (PDK1), which phosphorylates PDH, inhibiting this enzyme. The glycolytic switch is further supported by HIF1-α/HIF2-α, eventually promoting the expression of Glucose Transporter 1 (GLUT1) and Lactate dehydrogenase A (LDH-A) [8,9].

The established glycolytic landscape provide a support to the Pentose Phosphate Pathway (PPP), in turn playing an essential role in the cancer context (i) maintaining the redox homeostasis by glutathione production and (ii) providing pentoses to sustain cellular replication [10]. Interestingly, the oxidative branch of PPP initiated by the activity of Glucose-6-phosphate Dehydrogenase (G6PD) is preferentially used to synthetize ribose (56–66%) required for the cellular growth and of NADPH biosynthesis. Increased NADPH is indeed required to drive the reductive carboxylation of αketoglutarate (αKG) and fatty acid synthesis for rapid proliferation. Consequently, under FH-loss conditions PPP represents the central pathway for NADPH production [11].

### 1.2. Fumarate-Induced Succination

**Figure 2:**
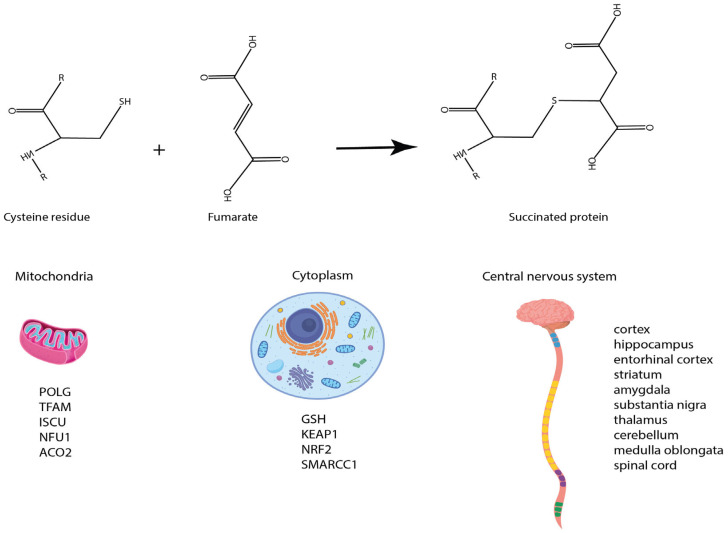
Representative scheme showing the reaction leading to succinated proteins. Fumarate reacts with cysteine residues via a Michael-like reaction resulting in a succinated cysteine residue. Mass spec analysis has reported an increase in succinated proteins in mitochondria, triggering their impairment, along with different proteins within the cytoplasm. Recently, an increase in succination has been also described in the central nervous system of aging individuals.

To overcome TCA-blockage, glutamine turns to be used by an anaplerotic reaction, obtaining αKG. The latter is essential for sustaining the NADH pool, which is required for the maintenance of ATP generation and Mitochondrial Membrane Potential (MMP). In FH-deficient cells, several works show a significant alteration of MMP. Particularly, Petros et al. revealed a higher MMP with the concomitant decrement of Proton Gradient (DpH), to keep the proton motive force constant; in this context, this not correlated with redox potential variation of the NAD^+^/NADH couple but rather the ubiquinone/ubiquinol couple, thus suggesting a possible involvement of the mitochondrial Respiratory Complex (RC) [8]. Accordingly, Crooks et al. showed that the alteration mitochondria-ultrastructure associated with MMP-variation in fumarate-accumulation state is correlated with impaired assembly of the RC [12]. In physiological conditions, such an event depends on the presence of the relative subunits encoded both by nuclear DNA and mtDNA. The authors showed that impaired assembly of complex was due to the significant loss of mRNA and protein abundance of complex subunits mtDNA-encoded, due to reduction in mtDNA content. Interestingly, the latter event was related to increased mtDNA mutation burden, due to loss of function of mitochondrial proteins, such as DNA Polymerase gamma (POLG) and Mitochondrial Transcription Factor A (TFAM), which are involved in replication and maintenance of nucleoids. In this context, the loss of protein function was attributable to fumarate-mediated succination (Figure 2) [12]. The latter is determined by the electrophilic nature of the fumarate which spontaneously reacts with the cysteine residues of many cellular proteins. The reaction yield is greatly enhanced by acidic pH environments as the typical tumor microenvironment (TME). Recently, around 182 different succinated proteins which are mainly located within mitochondria have been reported by Miglio and colleagues [13]. The detection of protein adducts has been originally reported in human erythrocytes and skin collagen, which is progressively succinated with aging [14]. Currently, it has been linked to different pathological models including diabetes, obesity, FH-deficient pathologies and Leigh syndrome. Besides the above-described POLG and TFAM, further succinated targets have been reported as regulatory proteins of iron metabolism and Fe-S cluster biogenesis, such as the Fe-S cluster assembly enzyme (ISCU) and Fe-S Cluster Scaffold Nfu1 (NFU1), whereby the cysteine residues involved in succination are critical for their enzymatic activity (Figure 2). This outcome leads to defects in the early and late steps of Fe-S cluster biosynthesis and deficiency of RC complexes. In this context, greater effects were observed for Complex I, harboring the largest number of Fe-S clusters. Complex II contains only three Fe-S clusters, and the observations that its activity is unaffected in FH-deficient state indicates that the defects in Fe-S cluster generation are milder compared with the effects of fumarate-driven inhibition, suggesting a marked activity reduction by fumarate in a concentration dependent manner [9]. Consequently, this could indicate that, while fumarate has no direct inhibitory effects on Complex I, it suppresses the activity of Complex II by inhibition of the product. Furthermore, the observed upregulation of SDH assembly factors could compensate for the decreased biogenesis of the Fe-S cluster, increasing the efficiency of their incorporation into the mature protein. Additionally, the succination of proteins involved in Fe-S cluster assembly potentially hinders aconitase (ACO2) activity (Figure 2) [8]. ACO2 can also be succinated. Physiologically, ACO2 catalyzes the citrate to isocitrate reaction. In contrast to mouse cells, where succination compromises the activity of ACO2 and leads mitochondrial dysfunction, in human cells it is unaffected, as indicated by its ability to perform reductive carboxylation [15,16]. Mitochondrial dysfunction induces tumor cells to rely on glutamine-dependent reductive carboxylation rather than oxidative metabolism, as the major pathway of citrate formation. Through this pathway, acetyl-coenzyme A is provided for lipid synthesis as well as intermediates needed to produce several macromolecular precursors, useful for sustaining the high growth rates of malignant cells. Glutamine-dependent reductive carboxylation represents the dominant metabolic pathway of tumor cells with mutations in FH and respiratory complexes [16].

As shortly mentioned above, protein succination has also been detected in diabetic models, where also glyceraldehyde-3-phosphate dehydrogenase has been reported as a possible succination target, leading to its inactivation in the hepatocytes of diabetic mice models [17]. Interestingly, these data were also mirrored in vitro on 3T3-L1 murine adipocytes. Once cultured in high glucose medium, supplied with 30 mM glucose, these cells increased the level of succinated proteins [18]. Similarly, streptozotocin-treated rats, used as a type 1 diabetes, as a db/db (leptin receptor deficient) model, showed a marked increase in protein succination in adipose tissue, skeletal muscle and urine [19]. Therefore, the current mechanism has been estimated to rely on an excess of nutrients orchestrating an increase in ATP/ADP and NADH/NAD+ ratio. These events trigger OXPHOS downregulation, eventually resulting in the accumulation of several metabolites, including fumarate [20]. Similarly, the loss of function of Ndufs4 in mice, eventually mirroring the Leigh syndrome, triggered a dysfunctional mitochondrial electron transport chain assembly. These mice were also characterized by an increase in the succination rate of several proteins [21]. 

Glutathione (GSH) can also be targeted by succination, leading to an increase in oxidative stress and consequent cellular senescence (Figure 2) [22]. Additionally, succinated and inactivated Kelch-Like ECH-Associated Protein 1 (KEAP1) cannot interact and cannot promote the proteasomal degradation of the transcription factor nuclear factor Erythroid 2 Like 2 (NRF2), which induces antioxidant response mitigating oxidative stress in tumor cells, thus promoting cell survival (Figure 2) [23,24]. 

An additional target of succination is the SWI/SNF-Related, Matrix-Associated, Actin-Dependent Regulator of Chromatin Subfamily C Member 1 (SMARCC1), involved in the formation of a tumor-suppressor complex, functioning as an ATP-dependent chromatin remodeling factor and regulator of the structure of the nucleosome (Figure 2). When inactivated by succination, it leads to a failure of the latter complex and alteration transcriptional profile, supporting the start of tumorigenesis [25]. Overall, succination promotes cancer cells in acquiring a more resistant phenotype, thus sustaining tumor growth and proliferation.

Further evidence highlighted how succination is progressively enhanced in the brain during aging. In neurons and glial cells, mitochondria represent the main energetic source thus providing a hint in investigating the increase in succination [26]. Accordingly, an enhanced level of protein succination was detected by mass spectrometry in the olfactory bulb, cortex, striatum, cerebellum and brainstem of mice [21]. Similarly, in the aged human cortex (occipital, temporal and frontal), hippocampus, entorhinal cortex, striatum, amygdala, substantia nigra, thalamus, cerebellum, medulla oblongata and spinal cord, the level of succination was increased (Figure 2) [27,28], overall providing a crosslink between aging, mitochondrial stress and protein post-translational modification.

Besides its role in driving protein modifications and the consequent metabolic switch, fumarate accumulation also shows a significant cytotoxic effect. Some studies have shown that fumarate accumulation leads to DNA damage, eventually reducing the overall cell viability, with minimal correlation with caspase 3/7-dependent apoptosis, thus suggesting the implication of additional pathways. This is supported by global DNA hypermethylation, whereby altered DNA methylation could affect genes involved in other apoptotic pathways [29]. To overcome its cytotoxic effect, fumarate is metabolized by several pathways, such as purine and urea cycles. In the first scenario, the overactivation of Purine Nucleotide Cycle (PNC) leads to high production of adenylosuccinate, via reverse reaction catalyzed by Adenylosuccinate Lyase (ADSL) [30]. The overactivation of urea cycle, on the other hand, triggers a reverse reaction, driving the synthesis of argininosuccinate from arginine and fumarate, thus leading to arginine depletion, eventually making the cancer cell greedier [31].

The plethora of changes mediated by fumarate accumulation might thus harbor a specific role in promoting tumor outcome and progression. For this reason, it might be crucial to further evaluate not only the changes affecting the metabolic landscape but also the ones acting on a further level of the cells accumulating fumarate. 

## 2. Fumarate-Mediated Epigenome Rewiring

Tumor microenvironment changes are often translated into epigenetics modifications affecting the whole cancer *milieu*. In this regard, several studies dissected the link between cell metabolism, cell fate and development. Readers are referred to some extensive publications which recently reviewed how metabolism determine stem cell fate [32,33,34,35]. Epigenetics is widely referred as a group of heritable changes rewiring gene expression without affecting the DNA sequence [36]. They concern histone modifications as methylation, hydroxylation, acetylation, lactylation, ubiquitination and SUMOylation modulating the chromatin architecture, along with the exchange of ca7nonical histone proteins with their non-canonical counterpart [37]. With this regard, several isoforms stepped on the front stage and gained further interest in the latest year. Our group and others have been focusing on the role of the largest H2A variant, macroH2A1, and its splicing variants, macroH2A1.1 and macroH2A1.2, in the establishment of senescence associated heterochromatin foci and stem cell renewal, both in solid and hematological malignancies [38,39,40,41,42,43,44]. All the epigenetics modifications are interconnected with changes in the cellular context, in turn determining the cellular fate itself. In this scenario, the importance of fumarate, as a metabolite reshaping the cellular epigenome, eventually establishing a crosstalk with changes in cellular metabolism, becomes undisputable. The reason behind this process is a consequence of the activity of those enzymes mediating epigenetic modification using several metabolites either as cofactors or substrates. Within this context, those chromatin-modifying enzymes using a metabolite having a physiological concentration close to or lower than their Km and Kd are the ones most sensitive to metabolic changes. On a separate level, epigenetic changes might also harbor a non-enzymatic nature, rather depending on the reactivity of the factors involved. To this extent, the detailed kinetic and thermodynamic properties must be addressed to gain further insights on how metabolic alteration might affect those epigenetic modifications.

Modifications to the epigenetic profile impact on the cellular state are also reported in the seminal work by Waddington, which in the 1940s introduced the concept of epigenetic landscape, serving as a blueprint describing the correlation between the epigenome and cellular state. From the energetic point of view, stable epigenotypes are represented by valleys, corresponding to a low energetic state, while summits correspond to the barriers to overcome for the transition from one epigenotype to another. In this context, changes in cellular metabolic state might affect Waddington’s epigenetic landscape (i) prompting the transition between two different epigenotypes by affecting specific chromatin modifications or (ii) completely reshaping Waddington’s landscape thus introducing a new stable epigenetic state. Regardless of the model, metabolic changes orchestrate an irreversible transition in the Waddington’s landscape, ultimately determining cellular fate. For this reason, despite the different models proposed, further studies are needed to be performed to investigate how the different metabolic conditions might reshape the epigenetic landscapes.

**Figure 3:**
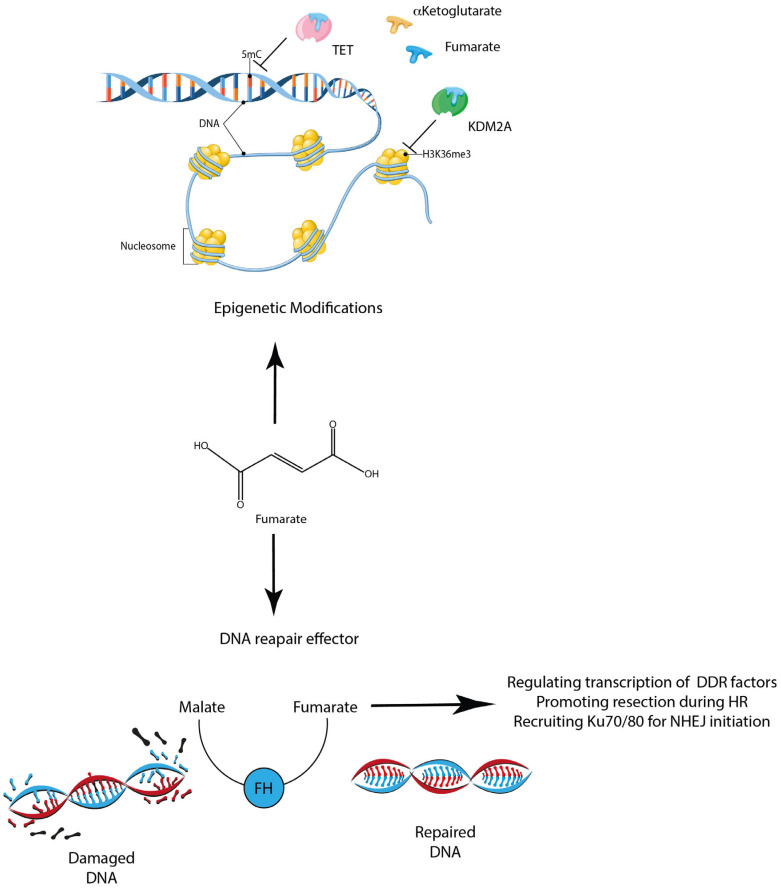
Scheme resuming fumarate roles in rewiring the cellular epigenetic profile and DNA damage repair machinery. Fumarate behaves as an antagonist of TET and KDM2A cofactor αKetoglutarate, in turn triggering their inactivation, thus leading to an increase in DNA and histone methylation index. FH activity, on the other hand, is required in the DNA-damaged site to promote the transcription of different DDR pathways. Furthermore, different evidence highlights how FH activity is crucial in promoting the resection step during HR and in recruiting the NHEJ-initiating complex Ku70/80.

Here, we focused on the epigenetic modifications orchestrated by metabolic changes, eventually determining tumor associated cell fate. These cells are usually characterized by a prominent glycolytic metabolism, although the OXPHOS contribution in regulating pluripotency must not be excluded. This finely tuned interplay affects the level of different metabolites, including the histone acetylases’ substrate acetyl-CoA, which has been reported to play an outstanding role in determining the human and mice stem cell pluripotency state [45]. The emerging role of fumarate as a metabolite determining cellular fate has also been recently reported within the tumoral context (Figure 3). Sciacovelli and co-workers prospected a scenario where fumarate might elicit epithelial-to-mesenchymal transition, the phenotypic switch associated with cancer initiation, invasiveness and metastasis, thus acting as an active oncometabolite [46]. Their work unveiled that FH-deficient cells increase Zinc Finger E-Box Binding Homeobox 1 and 2 (Zeb1 and Zeb2) expression, as a consequence of a decrease in Zeb-regulating miR-200 family. The authors report that miR-200 downregulation is related to a decrease in Ten-eleven Translocation (TET)-demethylating activity, resulting in fumarate accumulation, eventually competing with their cofactor αKetoglutarate [47] (Figure 3). Corroborating these data, the mir200ba429 regulatory region was found to be hypermethylated in FH-deficient cells, and its expression was eventually restored by fumarate supplementation. This evidence was also recapitulated in HLRCC patients, who were characterized by an increase in fumarate levels inhibiting TET activity within the tumor context but not in the proximal area [6]. Despite the multifaced effects triggered by fumarate accumulation, the authors suggest that one of the main outcomes is epigenetic EMT activation, in synergy with other metabolites serving for the same purpose, as succinate and 2-hydroxyglutarate possibly link EMT to mitochondrial dysfunction [6]. Furthermore, a crucial aspect of fumarate accumulation might be linked to its role in overcoming tumor growth factor b (TGFb)-mediated cellular growth arrest [48]. According to Chen and colleagues, FH is phosphorylated on Thr90 by p38, upon activation of the TGFb cascade. As a result, p-FH shifts to the nuclei. Here, following Notch activation, the Notch intracellular domain (NICD) promotes the interaction between CBF-1/RBPJ-κ (CSL) and p38-phosphorylated FH, eventually leading to the recruitment of the p-FH/CSL/p53/Smad complex [49]. The so-formed complex mediates the recruitment of p53 to p21 promoters. In this context, FH’s duty is to induce local accumulation of fumarate in order to inhibit lysine demethylase 2A (KDM2A)-H3K36me2 methylation, thus mitigating TGFb-induced cellular growth arrest, otherwise leading to cell senescence [48] (Figure 3). In this scenario, escaping cellular senescence might thus represent a strategy towards neoplasia development. Furthermore, within the cancerous context, cells keep their proliferation unaltered even upon nutrient-deficiency, probably bypassing the stress-induced pathways. Interestingly, FH might occur in this process by hampering Gene-Activating Transcription Factor 2 (ATF2) signaling. The latter is an activator of the protein 1 (AP-1) transcriptional factor family member, triggered by stress activated kinases c-Jun N-terminal Kinase (JNK) and p38. Under physiological conditions, AMPK phosphorylates FH and ATF2, eventually establishing a synergy enriching these two factors onto promoter regions of target genes [50]. Here, FH catalytic activity increases the local fumarate level, in turn inhibiting KDM2A, thus stabilizing H3K36me2 and eventually triggering the transcription of ATF2-targeted genes leading to cell growth arrest (Figure 3). However, in pathological conditions, as in cancer cells, one of the strategies to overcome this mechanism relies on aberrant post-translational modifications, such as O-GlcNAcylation of FH-AMPK phosphorylation sites, resulting in the disruption of FH-ATF2-mediated signaling thus promoting aberrant cell growth [50]. Besides its role in mediating tumor proliferation by directly affecting cancer cells, fumarate also reshapes the TME. It has been reported that fumarate might induce H3K4 methylation during b-glucan-mediated training of monocytes, eventually linking innate immunity stimulation with epigenetic and metabolic rewiring [51]. Interestingly, the administration of fumarate itself partially mirrors the b-glucan-induced trained immunity, in turn providing evidence for the outstanding role played by this metabolic intermediate in this context. Furthermore, the mechanistic hypothesis behind fumarate-induced trained immunity might be linked with (i) its role in regulating KDM5 bioactivity in synergy with 2-hydroxyglutarate and (ii) its inhibitory effect on HIF1a proteasomal degradation, an essential transcription factor in b-glucan-induced trained immunity [51]. Given this evidence, it is thus clear that fumarate might indeed represent a metabolite on the crosstalk between metabolism and epigenetic, eventually relying upon the mechanisms orchestrating cancer proliferation and immune escape. For this reason, further studies are needed to evaluate the potential of targeting fumarate metabolism to develop novel therapeutic strategies.

## 3. Fumarate-Mediated DNA Damage Repair

During the cellular lifespan, genome integrity maintenance is crucial to keep homeostatic conditions. Several diseases are indeed associated with the increasing amount of harmful DNA lesions [35]. Furthermore, misrepaired DNA damage is well known as a cancer-driving factor; when DNA is damaged, it can be indeed repaired by several DNA damage repair (DDR) pathways. These include a plethora of mechanisms where homologous recombination (HR) and non-homologous end-joining (NHEJ) represent the prominent members. The main difference between these two relys on the evidence showing that while HR operates an error-free DDR, in turn using the siter chromatid as a template for the synthesis of the new DNA strand, NHEJ usually induces mutations by just joining the DNA overhangs, resulting in DNA breakage [35,38]. The HR/NHEJ balance is strictly related to the chromatin status, which is in turn determined by the relative abundance of metabolites as Fumarate. As recently reported by Silas and colleagues, fumarate has indeed a striking role in Escherichia coli DDR [52]. This strains harbors three fumarase genes: class-I fumA and fumB and class-II fumC, which do not show any significant similarity, thus proving a different evolutionary origin. In this context, class-I fumarases participate in the DDR, while class-II fumarase has a role in the OXPHOS. The former plays a role in DDR on two levels: on one side it affects the global transcriptome profile of the fum-mull strains, in turn showing a decrease in DDR factor transcription (Figure 3); on the other hand, it hampers the αKG-dependent enzyme AlkB. This Fe-(II)/αKG–dependent dioxygenase catalyzes the direct reversal of alkylation damage to DNA, thus playing a pivotal role in DDR [52] (Figure 3). Overall, these data link metabolic dysfunction to efficient DNA repair, also involving fumarate accumulation. This step is strictly linked to the cytosolic FH translocation. Corroborating this, Yogev et al. reported an S. cerevisiae strain where the FUM1 gene was deleted from its former location on chromosome 16 and inserted within the mitochondrial genome. As a result, cytosolic fumarase was lost. Interestingly, this event enhanced S. cerevisiae sensitivity to HO, chemicals and y-irradiation-induced DSB. This effect was recovered upon fumarate supplementation, eventually proving that cytosolic FH enzymatic activity is essential for DDR. The same authors mirrored these data on human cell line models, where FH knockdown increased cell susceptibility to ionizing radiation and hydroxyurea-induced DSBs. Despite the evidence reporting the contribution of FH to DDR, its role in determining the prominence of a specific pathway is still debated. Leshets et al. reported that yeast fumarase is important within HR. During HR initial steps 50 to 1600, bases flanking the DSB region might be processed for resection [53]. The authors proved in this study that upon fumarase depletion the resection event was not taking place, thus prompting them to conclude that fumarase enzymatic activity is required for HR initial steps (Figure 3). Furthermore, a functional interaction between the DDR factor Sae2 and fumarase has been detected, in turn promoting the Mre11 dissociation following HR, thus determining its kinetic [54]. Further evidence supporting the linkage between Mre11 enzymatic activity and fumarate showed that Mre11 nuclease dead (mre11-nd) mutant cells recover their sensitivity to DNA damage agents upon fumarate supplementation. Since Mre11 is essential for the initial HR steps, this corroborates the scenario in which fumarase enzymatic activity has a pivotal role in this context [53]. Jiang and collaborators, on the other hand, showed that upon exposure to ionizing radiations, FH is recruited to histones, turning into part of the H2A.Z interactome. From the data reported by the authors, this step is essential for the accumulation of the Ku70/80 heterodimer, one of the central factors concurring for the DNA-dependent protein kinase (DNA-PK) holoenzyme formation, eventually being crucial in NHEJ (Figure 3). DNA-PK orchestrates FH phosphorylation on Thr236, triggering the binding of this newly formed complex to the double strand break (DSB) site in a NHEJ-specific fashion. The so-induced production of fumarate inhibits KDM2B-mediated H3K36 demethylation enhancing Ku70 binding, thus prompting DDR towards NHEJ [55]. 

## 4. Conclusions

Fumarate has been deeply studied as fundamental metabolic intermediate in the TCA cycle. However, in recent years several reports claimed that this molecule might be involved in cancer pathogenesis, thus prompting researchers to investigate the interconnection between metabolic disfunction and tumor progression, which was already at the center of the stage upon the Warburg postulate. As we discussed in this review, fumarate accumulation might indeed be the driver responsible for the rewiring of cellular epigenetic landscape. Therefore, the metabolic reorganization typical of malignant cells might work as a double edge sword, providing the substrates required during cellular proliferation and, at the same time, altering their transcriptomic profile by promoting several epigenetic modifications. Furthermore, recent reports determined the contribution of FH to DDR. As already stated, efficient repair is crucial for the keeping of the genome integrity, which once lost might turn out to be one of the prominent tumorigenesis drivers. Overall, this evidence supports the idea that fumarate might act at least upon two other different levels rather than the metabolic one: one concerning the cellular epigenome and the second affecting genetic integrity. For this reason, further studies are needed to deeply investigate how targeting fumarate metabolism might represent a novel strategy to counteract tumor onset and progression. 

## Abbreviations

TCA	Tricarboxylic Acid Cycle
FH	Fumarate Hydratase
MTS	Mitochondrial targeting sequence
HLRCC	Hereditary Leiomyomatosis and Renal Cell Carcinoma
IDH 1/2	Isocitrate Dehydrogenase 1 and 2
SDH	Succinate Dehydrogenase
OXPHOS	Oxidative Phosphorylation
AMPK	AMP-mediated protein kinase
PDH	Pyruvate Dehydrogenase
PHDs	Prolyl Hydroxylases
HIF1-α/HIF2-α	Hypoxia-inducible Factor 1/2-alpha
PDK1	Pyruvate Dehydrogenase Kinase 1
GLUT1	Glucose Transporter 1
PPP	Pentose Phosphate Pathway
G6PD	Glucose-6-phosphate Dehydrogenase
αKG	αketoglutarate
MMP	mitochondrial membrane potential
DpH	proton gradient
RC	respiratory chain
POLG	DNA Polymerase gamma
TFAM	Mitochondrial Transcription Factor A
TME	tumour microenvironment
ISCU	Fe-S cluster assembly enzyme
NFU1	Fe-S Cluster Scaffold Nfu1
ACO2	aconitase2
GSH	Glutathione
KEAP1	Kelch-Like ECH-Associated Protein 1
NRF2	Nuclear Factor, Erythroid 2 Like 2
SMARCC1	SWI/SNF Related, Matrix Associated, Actin Dependent Regulator of Chromatin Subfamily C Member 1
PNC	Purine Nucleotide Cycle
ADSL	Adenylosuccinate Lyase
Zeb1 and 2	Zinc Finger E-Box Binding Homeobox 1 and 2
TGFβ	tumor growth factor β
NICD	Notch intracellular domain
CSL	CBF-1/RBPJ-κ
KDM2A	Lysine Demethylase 2°
ATF2	Gene-Activating Transcription Factor 2
AP-1	Protein 1
JNK	c-Jun N-terminal Kinase
DDR	DNA damage repair
HR	Homologous Recombination
NHEJ	Non-Homologous End-Joining
DSB	double strand break
